# Mitigation of Airborne Contaminant Spread through Simple Interventions in an Occupied Single-Family Home

**DOI:** 10.3390/ijerph18115880

**Published:** 2021-05-30

**Authors:** Tanvir R. Khan, Danny S. Parker, Charles Withers

**Affiliations:** FSEC Energy Research Center, University of Central Florida, Cocoa, FL 32922, USA; DParker@fsec.ucf.edu (D.S.P.); chuck@fsec.ucf.edu (C.W.)

**Keywords:** SARS-CoV-2, ventilation, isolation zone, containment, particulate matter

## Abstract

Historically, reducing aerosol-based transmission of respired viruses in indoor environments has been of importance for controlling influenza viruses and common-cold rhinoviruses. The present public health emergency associated with SARS-CoV-2 makes this topic critically important. Yet to be tested is the potential effectiveness of simple interventions that create an isolation zone (IZ) for a suspected/confirmed sick or sensitive person requiring quarantine. The intent in existing homes is to find a practical means to mitigate exposure to airborne contaminants. In creating an IZ in an occupied single-family home in the study, four simple strategies were tested. The test configurations were: (1) IZ windows closed with IZ bathroom exhaust ventilation fan off, (2) IZ windows closed with IZ exhaust fan on, (3) IZ window open with IZ exhaust fan off, and (4) IZ window open with IZ exhaust fan on. Incense-generated fine particulate matter (PM_2.5_) was used as a marker for virus transmission. The measured transfer of PM_2.5_ from the IZ into the main zone (MZ) of the house enabled us to determine the relative effectiveness of four containment strategies. Collectively, the data from pressure differential (across zones) and PM_2.5_ measurements suggested that the best containment strategy was achieved through continuously operating the bathroom exhaust fan while keeping the windows closed in the IZ (configuration 2). Interventions using open windows were found to be less reliable, due to variability in wind speed and direction, resulting in an unpredictable and sometimes detrimental pressure differential in the IZ with reference to MZ. Our findings strongly suggest a simple IZ exhaust ventilation strategy has the potential for mitigating the risk from the airborne spread of contaminants, such as SARS-CoV-2.

## 1. Introduction

Historically, reducing airborne transmission of respired viruses in indoor environments has been of importance for controlling influenza viruses and common-cold rhinoviruses. With the outbreak of the severe acute respiratory syndrome coronavirus 2 (SARS-CoV-2) pandemic, health risks posed by their airborne transmission in indoor environments have gained significant attention in scientific communities [1,2,3,4,5]. During breathing, speaking, sneezing, and coughing, infected individuals exhale both virus-laden droplets (>5 to 10 µm) and aerosols (<5 µm) [1,6]. Viruses in aerosols can remain airborne for hours, and be inhaled deep into the lungs [7,8]. While the diameter of SARS-CoV-2 is in the range of 0.06 to 0.14 µm [9], they tend to be associated with aerosols in the size range of 1 to 4 µm and are readily transported by air [10,11]. Thus, making effective containment of this virus challenging [12].

Despite uncertainties regarding the relative importance of the various transmission modes, existing evidence strongly suggests that SARS-CoV-2 is primarily transmitted indoors by aerosols [4,5,12,13]. The public health emergency associated with SARS-CoV-2 makes controlling airborne transmission of respired viruses in indoor environments critical, especially in poorly ventilated indoor environments. Sharing indoor space in the presence of infected individuals has been a major risk factor in the transmission of SARS-CoV-2 [1,3]. To mitigate the risk for the population in indoor environments, methods of at-home isolation become highly desirable since further home infections are very common in shared occupancy, once a single household member is infected [14,15].

The current body of knowledge, therefore, indicates the importance of isolating an infected person from other assumed to be non-infected persons in the same residence. Then the obvious question is if one member of the household has suspected or confirmed infection or required to be quarantined, how would one best mitigate chances for additional infection within the household? While it is common to locate an infected household member in a separate bedroom, how effective is this strategy, and what factors may influence its effectiveness?

ANSI/ASHRAE/ASHE Standard 170-2017 requires that healthcare facility isolation rooms or zones be continuously maintained at 2.5 Pascal pressure lower than adjacent spaces [16]. This pressure requirement provides some guidance for IZ in residences. Ventilation is another important factor needed to reduce airborne viral load. This is a difficult matter to control in residences as most do not have whole-house mechanical ventilation, and those that do are typically not adequately ventilated [17,18,19]. Ventilation strategies available to most residents are open windows and maybe bathroom exhaust fans.

Qian and Zheng [20] specifically identified the importance of ventilation control for infectious agents that present as aerosols. Allen and Marr [1] focused on a multi-faceted approach to ameliorate infection potential including ventilation and negative pressure in infected zones. To reduce the health risks for airborne transmission in residential buildings including single-family homes and apartments, practical measures such as segregating infected individuals, opening windows and doors, and using portable air-cleaning devices are commonplace [5]. However, the concept of utilizing negative pressure or depressurization while creating an isolation room or IZ in an occupied single-family home as a practical approach to protect the rest of the people in the home has not been tested yet, to the best of our knowledge.

In this study, four containment controls were tested in an occupied single-family home under various operating conditions. These interventions were designed based on the utilization of a bathroom exhaust fan for pressure control while keeping the IZ door to MZ closed, and the IZ window(s) both closed and open. The primary metric used in this study to evaluate potential containment effectiveness was differential pressure (dP) established by ASHRAE Standard 170, which calls for IZ with respect to (w.r.t.) the MZ of the house to be at least 2.5 Pascal lower. This pressure differential requirement was the primary basis of comparing the efficacy of the four containment strategies.

Incense smoke-generated fine particulate matter (PM_2.5;_ aerosols with aerodynamic diameters 2.5 micrometres and smaller) in the IZ was used as a marker for virus transmission as well as supplementary means of observing containment. The rationale for selecting PM_2.5_ as a marker for virus transmission is described in detail in Section 2.3. The overarching objective of the study is to elucidate how various ventilation strategies influence the containment potential of contaminants in the IZ. Based on this evaluation, recommendations for simple, low-cost, and do-it-yourself (DIY) interventions for existing homes are provided to mitigate exposure from the airborne transmission of contaminants.

## 2. Methods

### 2.1. Description of Test Space

**Figure A1:**
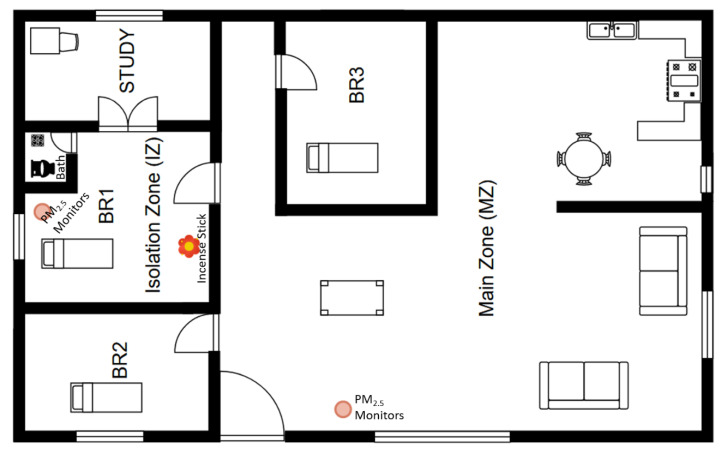
Generic floor plan of the house (not drawn to scale), showing various rooms, and locations of PM_2.5_ monitors.

An occupied single-family home located in Cocoa Beach, Florida, USA was used to conduct the experiments in May 2020. The floor area of the house was ~185 m^2^ with an average ceiling height of 2.4 m. The house was built on a slab foundation. The MZ was denoted as the living room space, which was adjacent to the IZ by a short hallway. The MZ typically had very low occupancy during the test periods. The master bedroom was used as an example isolation room or IZ, having a 4 m × 4 m space with two well-sealed windows on the east side of the house. There are three doors in the IZ. The first one was a 1.2 m × 1.5 m door (with a 0.95 cm undercut) providing access between the IZ and MZ. The second door (1.4 m wide with a 0.32 cm undercut) opened to an adjacent study space having no other access to the home. These two doors remained closed during the experiments. The third door was between the IZ and a small bathroom with a ceiling exhaust fan, which remained open during the experiments. There were no windows in the bathroom. A generic floor plan (Figure A1) on the test house is included in Appendix A.

The bedroom area and other parts of the home were cooled by ductless mini-split heat pumps with no central ducted air, and there was no influence from mechanical forced air from one room to another. Therefore, there was no pressure differential created during space conditioning and no dilution of test-generated PM_2.5_ in the IZ. This home with no central forced-air system is more likely to contain air movement across the room than homes with a central forced-air system. The bathroom centrifugal exhaust fan (FV07-VQL4, Panasonic North America, Newark, NJ, USA) had a manufacturer-stated nominal fan flow rate with no static pressure of 80 cubic feet per minute (CFM) or 38 L·s^−1^.

### 2.2. Measurements of Exhaust Fan Flow Rate and Differential Pressure

An Energy Conservatory Exhaust Fan Flow Meter (equipped with a digital manometer Model DG-700, The Energy Conservatory, Minneapolis, MN, USA) was used to measure the operational exhaust fan airflow rate (accuracy ±10%). Three repeated flow measurements were conducted with each series of measurements conducted with both IZ windows open and both closed. The dP across the zones was measured using the model DG-700 precision digital manometer with repetitions of three measurements each taken as ten-second averages.

### 2.3. Smoke-Generated PM_2.5_ as a Marker of IZ Containment Potential

The sizes of most respiratory viruses (e.g., rhinovirus, adenovirus, and SARs-CoV-2) are very small (i.e., 0.3–0.12 µm) [21]. Existing evidence suggests that airborne particles could play an important role in the transmission of respiratory viruses such as SARS-CoV-2 [22,23]. Early data suggest PM_2.5_ could act as a carrier for SARs-CoV-2 [24]. The virus-laden particles may remain suspended in the air for prolonged periods, especially particles having a smaller diameter with a low removal rate from air to surface, while the larger particles are subject to fast settling on surfaces [25,26].

Several recent studies reported that indoor particulate matter generated from tobacco smoking, incense burning, laser printers, etc. could facilitate the transmission of SARS-CoV-2 in indoor environments [27,28,29]. Given the limited knowledge about the production and airborne behavior of infectious respiratory aerosols, Prather et al. [7] suggested the airborne behavior of SARS-CoV-2 virions contained in aerosols is comparable with exhaled cigarette smoke as they both contain submicron particles and are likely to follow similar flows and dilution patterns.

Collectively, evidence from these studies led us to choose PM_2.5_ as an adequate marker for virus transmission potential and for evaluating the effectiveness of containment strategies. The source of PM_2.5_ was smoke generated from incense sticks. The incense used is ordinary Japanese incense (Mainichiko). Each stick was 14 cm in length with a dry weight of 0.3201 g (median of six measured samples). The measured mass of the ash after a complete burn was 0.0262 g, implying that approximately 0.3 g becomes airborne during combustion.

### 2.4. Indoor Air Quality Measurement

To measure time-resolved PM_2.5_ concentrations, both research-grade and low-cost indoor air quality monitors were used. Two MetOne BT-645 units (research-grade monitors) and two AirVisualPro (AVP) units (low-cost commercially available sensors) were deployed inside the house. The working principle of the BT-645 monitors is based on forward light scattering laser nephelometry. Factory suggested concentration range for these units is 0 to 100 mg·m^−3^ (0 to 100,000 µg·m^−3^). Their sensitivity and accuracy are 1 µg·m^−3^ and ±5%, respectively. During the experiments, the temporal resolutions of the MetOne units were 60 s with a calibrated airflow rate of 2 L·min^−1^. For PM_2.5_, these units can measure particle size ranging from 0.6 to 2.5 µm. The sensors in the AVPs were factory-calibrated through an automatic process in a controlled chamber for distinct particle size ranges. Their reported concentration range is 0–1798 µg·m^−3^. For PM_2.5_, the AVPs can measure particle size ranging from 0.3 to 2.5 µm. For all experiments, the temporal resolution of the AVPs was 10 s.

### 2.5. The First-Order Decay Rate of PM_2.5_ in IZ

To provide a relative assessment of how rapidly the viral load in the IZ could be decreased under various interventions, first-order rate constants were calculated from the observed PM_2.5_ concentrations. The decay period was used to calculate the loss rates. The start of the decay period was taken as the time when peak concentration was reached, and the end of the decay was identified as the last three consecutive intervals with zero or negative change following the peak. This method of calculating decay rates for PM_2.5_ is described in detail by Chan et al. [30]. The loss rate was calculated by fitting the PM_2.5_ time series to either 1st hour of the decay period or the entire decay period if it was shorter than 1 h. We fitted the concentration data assuming a pseudo-first-order rate equation (Equation (1))

(1) $$\;\ln\frac{C_{t}}{C_{0}}=-kt$$

where $C_{t}$ is the concentration (µg·m^−3^) at time *t* (min) following the peak concentration $C_{0}$ (µg·m^−3^) and k are the first-order decay or loss rate constant (min^−1^).

## 3. Results

**Figure 1:**
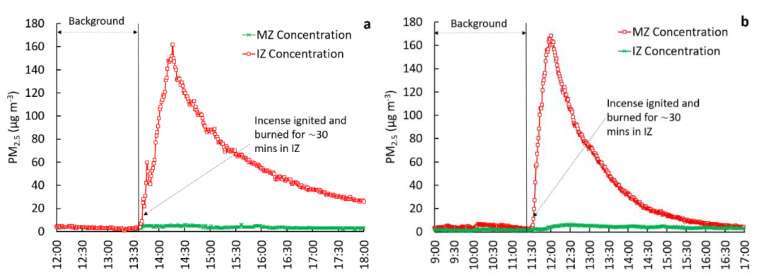
Comparison of PM_2.5_ concentrations in the IZ and MZ under the IZ windows closed, bath exhaust ventilation off (Test 1) scenario with trial 1 on the left (**a**) and trial 2 on the right (**b**).

**Figure 2:**
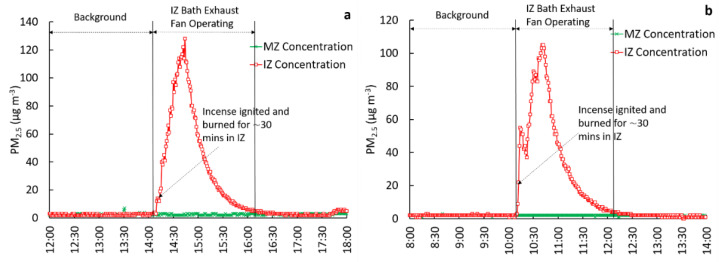
Comparison of PM_2.5_ concentrations in the IZ and MZ under IZ windows closed, bath exhaust ventilation on scenario (Test 2) with trial 1 on the left (**a**) and trial 2 on the right (**b**).

**Figure 3:**
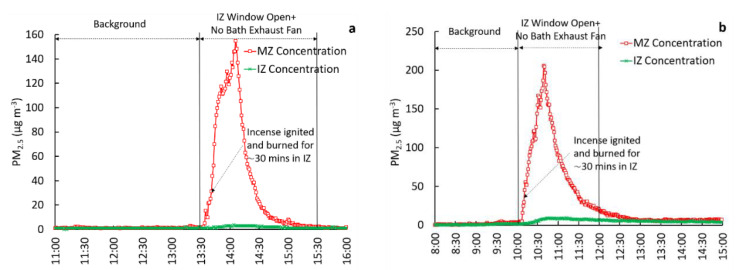
Comparison of PM_2.5_ concentrations in the IZ and MZ under one IZ window open, exhaust ventilation off scenario (Test 3) with trial 1 on the left (**a**) and trial 2 on the right (**b**).

**Figure 4:**
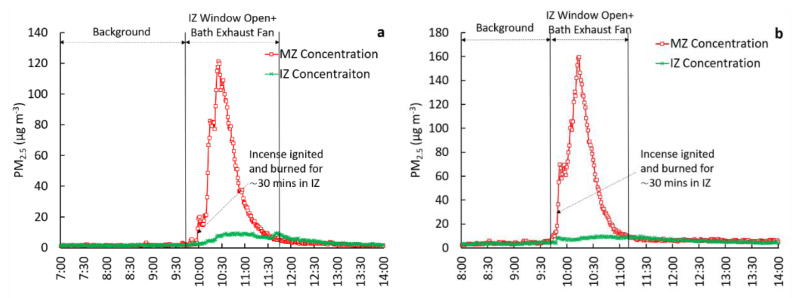
Comparison of PM_2.5_ concentrations in the IZ and MZ under one IZ window open, exhaust ventilation on scenario (Test 4) with trial 1 on the left (**a**) and trial 2 on the right (**b**).

To evaluate containment in the IZ, concurrent measurements of PM_2.5_ in the IZ and MZ were compared for four test configurations: (1) IZ windows closed, bath exhaust ventilation off (reference case), (2) IZ windows closed, bath exhaust ventilation on, (3) IZ window open, bath exhaust ventilation off, and (4) IZ window open, bath exhaust ventilation on. The door from the IZ to MZ remained closed while testing these scenarios. To evaluate the relative effectiveness potential of each configuration, interventions 2, 3, and 4 were compared to reference case 1. As part of the evaluations, the observed PM_2.5_ concentration ratios between zones, particle loss rates within the IZ, and pressure differential measurements across the zones were used. We note that in Figure 1, Figure 2, Figure 3 and Figure 4, the scaling of Y-axes varies, primarily due to challenges associated with maintaining identical peak concentrations in repeated trials. Factors such as natural infiltration (through building cracks, window cracks, etc.), are uncontrollable factors that tend to be highly variable daily, affecting the magnitude of peak PM_2.5_ concentrations in the IZ. All tests were conducted in an occupied house. Therefore, the start and end times of the experiments varied, allowing for the convenience of the occupants. As a result of this variability, the start and end times shown in the X-axes also varied between figures.

### 3.1. Exhaust Fan Flow Rate, Zone Differential Pressures, and PM_2.5_ Test Results

The IZ bathroom fan exhaust flow rates, both with windows open and closed were stable at 32–33 CFM with a mean value of 32 CFM or 15 L·s^−1^. The differential pressure (dP) in the IZ w.r.t. MZ of the house for four test configurations are listed in Table 1. The near-neutral pressure difference (dP~0 Pa) was observed when no controls were applied (reference case 1). In test configuration 2, modest depressurization was achieved (dP = −0.4 Pa) by operating the bathroom exhaust fan.

With windows open, local wind conditions may transport air back and forth between zones in the house depending upon wind speed and direction. As can be seen from Table 1, test configurations 3 and 4, both of which involved open windows, were unable to create depressurization in the IZ w.r.t MZ. Instead, the IZ was positively pressurized w.r.t. MZ (dP = +1.4 Pa and +1.5 Pa, respectively), thereby demonstrating inadequate containment pressure under the available weather conditions. It is also worth noting that the IZ exhaust fan with an open window (Test 4) did not have adequate flow to depressurize IZ.

Ratios of mean PM_2.5_ concentrations measured during an intervention (int) and the background (bk) period, denoted as MZ(int)/MZ(bk) and MZ(int)/IZ(int) were used to evaluate the effectiveness containment potential. For strong IZ containment, MZ(int)/MZ(bk) ≅ 1 is desirable, indicative of little to no transfer of contaminant from IZ to MZ during an intervention. In other words, for MZ(int)/MZ(bk) ≅ 1, PM_2.5_ concentrations in the MZ were identical before and after the intervention. Inter-comparison of the concentration ratios, MZ(int)/IZ(int), is also useful in evaluating the effectiveness of containment in the IZ. Generally, a lower MZ(int)/IZ(int) value (close to 0) for PM_2.5_ denotes a strong IZ containment.

However, operating conditions of the IZ during an intervention may affect the decay rate of injected aerosols, which could result in a rapid decrease in concentrations in the IZ. In such cases, even a small increase in concentrations in MZ could result in large MZ(int)/IZ(int) ratios, which could infer containment that is worse than actual. Considering this caveat, the effectiveness of an intervention measure was examined using both MZ(int)/MZ(bk) and MZ(int)/IZ(int) values in the context of pressure differentials (e.g., IZ dP), while determining the best and worst-case containment scenarios listed in Table 1.

In the IZ, the PM_2.5_ loss process is assumed to be primarily controlled by the operation of the bathroom exhaust fan and/or opening of windows and deposition. Therefore, a relative comparison of the loss rate (*k*) values (Table 1) provides an assessment of how fast PM_2.5_ was removed from the IZ under any given intervention or lack thereof. A higher *k*-value denotes a faster removal of contaminants from the IZ, while a smaller *k* value indicates slower removal and potential build-up of contaminants in the IZ.

### 3.2. Evaluation of Containment Potential in the IZ

PM_2.5_ was measured as a means to help determine containment effectiveness relative to the reference case (Test 1). It was a surrogate for the aerosolized virus. PM_2.5_ is also a known potential health hazard and as such offered an opportunity to consider not only viral containment but also pollutant containment of common household pollutants.

#### 3.2.1. IZ Windows Closed, Bath Exhaust Ventilation off (Test 1)

This test scenario represents the reference case where no intervention was applied. The door between the IZ to MZ was kept closed during the experiments. The results from two repeated experiments representing this case are shown in Figure 1. The background period, indicated in these figures and subsequent figures, refers to the time duration before the incense ignition. The duration of the background period varied from test to test and ranged from 1.5 to ~2.5 h. This variability is primarily due to the convenience of the occupants living in the house, which determined the start of the incense ignition time for each test.

Figure 1 shows that the background concentrations in both IZ and MZ were very low (mean < 5 µg·m^−3^). After the incense ignition, which burned for ~30–35 min, PM_2.5_ concentrations increased rapidly in the IZ, reaching a maximum concentration of ~160 µg·m^−3^. In the absence of any intervention such as an exhaust and/or window ventilation, the observed decay in concentrations in the IZ was attributed to loss due to particle deposition on surfaces within the IZ room as well as a small amount of naturally induced infiltration from internal air buoyancy (the wind).

Because the central HVAC system in the house was non-operational, particle losses in the HVAC systems did not occur. In the MZ, the concentration ratios (MZ(int)/MZ(bk)) ranged from 1.4 to 2.6, suggesting a modest increase (an increase by a factor of ~2) in PM_2.5_ concentrations in the MZ as compared to the background concentrations, while the incense was burning in the IZ. The mean concentration ratio between MZ and IZ (for t = 2 h following incense ignition in the IZ) was 0.05. The calculated *k* values for Test 1 trial 1 and trial 2 are 0.7 and 0.8 h^−1^, respectively.

#### 3.2.2. IZ Windows Closed, Bath Exhaust Ventilation on (Test 2)

This test configuration included the control using an exhaust ventilation fan located in the bathroom within the IZ and was repeated four times. Results from two of the four experiments are shown in Figure 2.

The measured background concentrations in the IZ and MZ were very low and comparable to one another (Figure 2). For all tests performed under this configuration, the start of the incense burn period coincided with the bath fan exhaust start time. The fan was operational for two hours. Following the peak concentrations and complete burnout of the incense sticks, the observed concentration profiles of PM_2.5_ in the IZ exhibited a rapid decline, caused by a combination of factors.

The most dominant factor was due to the exhaust fan, which increased IZ ventilation as well as transported some particulate outdoors. The other loss factor was due to particulate deposition. During the IZ fan operational periods, the mean MZ concentrations were 2.4 and 2.0 µg·m^−3^ as compared to the mean background concentration of 2.5 and 2.0 µg·m^−3^ for trials 1 and 2, respectively. This finding suggests that during exhaust ventilation fan operation, there was little to no transfer of contaminant from the IZ to MZ.

In contrast, for Tests 1 and 2, the mean IZ concentrations were 43.2 and 37.6 µg·m^−3^ when the bath fan was operational, respectively. Collectively, for all four trials, the mean concentration ratio (MZ(int)/MZ(bk)) was 0.9 (Table 1), suggesting a slight decrease in PM_2.5_ concentrations in the MZ during the intervention as compared to the background concentrations. The mean concentration ratio between MZ and IZ (for t = 2 h following incense ignition in the IZ) was 0.06 (Table 1). The mean *k* value for all tests was calculated as 2.6 h^−1^. The MZ(int)/MZ(bk) values ranged from 0.7 to 1.0 (Table 1), suggesting that _the_ concentration in the MZ did not increase during the simultaneous emissions of PM_2.5_ and bathroom exhaust fan operation in the IZ. The modest differential pressure data (dP = −0.4 Pa) also supports this observation as the negative pressure in the IZ restricted transfer of air containment from the IZ to MZ.

#### 3.2.3. IZ Window Open, Bath Exhaust Ventilation off (Test 3)

In this test configuration, one window in the IZ was left open while the bath exhaust fan remained off. The test was repeated three times. The results from the two trials are shown in Figure 3. The background concentrations in the IZ and MZ in both trials remained very low and were identical. During the incense-smoke generation and window open period (t = 2 h), the mean IZ concentrations were 40.2 (Figure 3 trial 1) and 74.6 µg·m^−3^ (Figure 3 trial 2), respectively. Figure 3 shows that concentrations in the MZ increased modestly during the testing periods compared to the background concentrations, which is an indicator of inadequate containment.

Together, for all three tests, the mean concentration ratio (MZ(int)/MZ(bk)) was 2.7 (Table 1), suggesting an increase in PM_2.5_ concentrations in the MZ during the intervention. The differential pressure data (dP = +1.5 Pa) also supports the observation of inadequate containment since the IZ was at positive pressure instead of negative pressure w.r.t. MZ. The mean concentration ratio between MZ and IZ (for t = 2 h following incense ignition in the IZ) was 0.07 (Table 1). The mean *k* value for all tests was calculated as 3.6 hr^−1^.

#### 3.2.4. IZ Window Open, Bath Exhaust Ventilation on (Test 4)

This test configuration involved operating the bathroom exhaust fan while keeping one window in the IZ open. Three trials were performed under this configuration. The concentration time series for two such experiments are shown in Figure 4. For the background periods, concentrations in the IZ and MZ were very low and identical. However, concentrations in the MZ increased while the incense was emitting PM_2.5_ in the IZ, and the exhaust fan was operational with the IZ window open.

Collectively, for Tests 2–4, the mean concentration ratios (MZ(int)/MZ(bk)) were 3.5 (Table 1), suggesting a strong increase in PM_2.5_ concentrations in the MZ during the intervention. The mean concentration ratio between MZ and IZ (for t = 2 h following incense ignition in the IZ) was 0.15 (Table 1). The mean *k* value for all tests was calculated as 3.0 hr^−1^.

The results indicate that controls applied in this test configuration were unable to contain PM_2.5_ within the IZ. The operation of the exhaust fan with one window open created overall positive pressurization in the IZ of 1.5 Pa w.r.t. MZ. This further demonstrated that the positive pressure in the IZ facilitated the transfer of air from the IZ to MZ thereby resulting in incomplete containment. Collectively, the results indicate that the direction (towards versus away from the IZ room) and wind speed can have a major influence on containment potential and transfer of contaminants from one zone to another.

## 4. Discussion

Based on the pressure differential and particulate containment results of four different operational scenarios, Test 2 demonstrated a better potential for isolating the IZ air from MZ air. Test 2 ran a bathroom exhaust fan located in the IZ continuously while keeping the IZ windows and IZ to MZ door closed. The PM_2.5_ concentration ratios (Table 1) also support the efficacy of this intervention. Of the four test configurations, the lowest value of MZ(int)/MZ(bk) was achieved through the Test 2 method. Comparison between MZ(int)/IZ(int) values listed in Table 1 could also be used to determine the relative effectiveness of various controls. Among three test interventions, the lowest and highest MZ(int)/IZ(int) values (0.06 and 0.15), were obtained for Tests 2 and 4, respectively.

A lower value of MZ(int)/IZ(int) indicates better IZ containment (e.g., 0.06 for Test 2), while a large value (e.g., 0.15) for Test 4 indicates weak IZ containment. The loss rate values are useful in assessing how rapidly aerosolized contaminants are removed from the IZ. However, these values alone should not be used to evaluate the effectiveness of IZ containment strategies.

As can be seen from Table 1, Test 3 resulted in the highest *k* value, yet controls applied in this test were unable to create depressurization in the IZ, resulting in a transfer of containment to MZ. Any intervention using open window(s) (with or without an exhaust fan) is highly dependent on outdoor wind conditions, thus making such interventions unreliable.

The results presented here are based on a single home with a bathroom exhaust fan flow of only 32 CFM (15 L·s^−1^). While this is not unusually low, it is substantially lower than the code minimum required intermittent local ventilation exhaust airflow rate for bathrooms (50 CFM or 24 L·s^−1^) (ASHRAE 62.2). Homes with more airtight construction and higher exhaust fan flow rates in the IZ would have greater depressurization and even better containment potential than the home in this study. However, as many exhaust fans operating in homes may be producing low flow rates, these particular tests demonstrate potential effectiveness even in sub-optimal circumstances.

In the U.S., certain types of healthcare facilities use negative-pressure airborne infection isolation rooms (AIIRs) for patients with airborne transmissible infections (Miller et al., 2017). To ensure virus containment in an AIIR, the pressure in AIIRs with reference to an external zone such as a hospital corridor is recommended to be −2.5 Pa (ASHRAE Standard 170, 2013). The existence of low flow (<50 CFM or 24 L·s^−1^) exhaust fans are likely to be common in many older homes, providing less potential containment than fans with around 100 CFM (47 L·s^−1^) or more.

However, the use of an available low-flow fan would still be better in an effort to limit the airborne spread of viruses in existing homes than not operating it at all. While open windows provide the benefit of dilution for contaminants through increased air exchange, achieving adequate IZ depressurization and containment by opening windows is unpredictable since containment can be intermittent depending upon wind speed and direction. Therefore, from a reliability standpoint, any containment approach/control that relies solely upon natural infiltration of air through IZ open windows is not recommended, although results indicated better than sealed results.

There are several caveats within the study. This evaluation was conducted in an occupied home with three occupants, which necessitated some compromise on methods. For instance, a longer period of particulate production might be advisable, but 30 min of pleasant stick incense was considered acceptable by the household involved. The house itself had several limitations that should be understood relative to its representativeness of housing in North America. The greatest difference was that the space conditioning system consisted of room-by-room ductless multi-split heat pumps rather than the conventional central air ducted heat pump. These could dramatically impact zonal pressure differentials as well the impact of the bathroom exhaust fan being used as to whether a negative pressure would be maintained across the doors. The authors point out, however, that ductless cooling systems, while atypical in the U.S., are the most common system types in the rest of the world. Similarly, while central heating with blowers is typical in the U.S., hydronic heating systems are most common around the rest of the world. Our results are relevant to those systems as well.

Future work: The preliminary results obtained through these exploratory experiments strongly support the idea of creating a residential isolation space to protect the healthy occupants in the house from a contagious person. Given the challenges of performing these experiments in an occupied home, future work will focus on systematically testing additional control measures in a laboratory home. A house with a central heating/cooling system would offer an opportunity to evaluate additional likely test scenarios and challenges for North American homes. A few examples of future test scenarios could include various HVAC configurations, utilization of bathroom and/or portable window fans for pressure control, interventions including closing the IZ door and/or closing/opening IZ window(s), sealing off supply air grilles in the IZ, and blocking off any jump/transfer return air grilles in the IZ. A portable room air conditioner with the exhaust duct installed in one of the IZ windows could also be used as an alternative to a window exhaust fan or bathroom exhaust fan.

## 5. Conclusions

During the SARS-CoV-2 pandemic, the need emerges to arrange isolation of suspected/confirmed infected or vulnerable household members in a way that can reduce the danger of spreading the infection to other persons in the household. Effective interventions in residences need to be quick, simple to implement, and low cost. The results from this exploratory research suggest potential effective isolation control can be achieved through the use of isolation zone air exhausted to the outdoors with the isolation zone doors and windows closed.

Adequate depressurization can be accomplished by either using a continuously running bathroom exhaust fan or window fans, which can be inserted into a single-hung window that exhausts air outdoors. This strategy reduces concentrations in the isolation zone and helps limit contaminated air moving from the isolation zone to indoor adjacent zones. While the negative pressure produced in the isolation zone of this study was weaker than desired, it still demonstrated better isolation than three other simple control options tested. The weak depressurization is due to a bathroom fan with low airflow under 50 CFM (24 L·s^−1^). This is common in existing U.S. homes and points out a common limitation. Greater depressurization can be accomplished by replacing the fan with one that can move at least 100 CFM (48 L·s^−1^) in-situ or simply adding a fan into one of the isolation zone windows.

It is also important to limit air pathways from the isolation zone into adjacent occupied zones. Seals around doors and blocking off central heating and cooling ducts into the isolation zone are a few options to improve tightness. A tighter zone will become more depressurized than a leakier one with the same exhaust airflow rate.

Limiting the spread of infectious diseases requires several measures that address different modes of transport. This project focused solely on limiting the transport of aerosolized pollutants from an isolation zone to the adjacent main zone of a specific occupied home. It is intended to complement other important public health measures such as wearing masks, frequent hand and surface cleaning, as well as using evidence-based air cleaning approaches such as filtration in the isolation zone to potentially remove particles that might carry the infectious virus.

## Figures and Tables

**Table 1:** Statistical Summary of Pressure Differential (dP) and PM_2.5_ Test Results.

Configuration	Trial	Mean dP (Pa)	PM_2.5_ Ratio ^1^ MZ (int)/ MZ (bk)	Mean ^2^ MZ (int)/ MZ (bk)	PM_2.5_ Ratio ^3^ MZ (int)/ IZ (int)	Mean MZ (int)/IZ (int)	PM_2.5_ Loss Rate in IZ, *k* (hr^−1^)	Mean Loss Rate in IZ, *k* (hr^−1^)
1. Window Closed+Exh Fan Off	1	~0	1.4	2.0	0.05	0.05	0.7	0.8
	2		2.6		0.05		0.8	
2. Window Closed+Exh Fan On	1	−0.4	1.0	0.9	0.06	0.06	2.4	2.6
	2		1.0		0.05		2.4	
	3		0.7		0.06		3.0	
	4		1.0		0.05		2.4	
3. Window Open+Exh Fan Off	1	+1.4	1.9	3.5	0.05	0.07	3.6	3.2
	2		7.3		0.09		2.0	
	3		1.2		0.07		3.9	
4. Window Open+Exh Fan On	1	+1.5	4.2	2.7	0.19	0.15	2.8	3.0
	2		2.2		0.18		3.2	
	3		1.8		0.08		3.1	

^1^ int denotes intervention period; bk denotes background period. ^2^ MZ(int) denotes mean PM_2.5_ concentrations in the MZ during an intervention (followed by the background period). This term represents the mean concentration in the MZ during the intervention period (t = 2 h). MZ(bk) denotes mean PM_2.5_ concentrations in the MZ during the background period. ^3^ IZ(int) denotes mean PM_2.5_ concentrations in the IZ during an intervention. This term represents the mean concentration in the IZ during the intervention period (t = 2 h).

## Data Availability

The data presented in this study are available on request from the corresponding author.
